# Research Advances and Biological Features of IgA for Immune-Related Diseases

**DOI:** 10.3390/ijms27146471

**Published:** 2026-07-21

**Authors:** Han Guo, Xinying Li, Fenghao Peng, Jijun Yu, Jing Wang, Longlong Luo, He Xiao, Guojiang Chen, Chenghua Liu, Jiannan Feng, Chunxia Qiao

**Affiliations:** State Key Laboratory of National Security Specially Needed Medicines, Beijing 100039, China; hanuull@163.com (H.G.); lychee526@126.com (X.L.);

**Keywords:** IgA, therapeutic antibody, inflammatory disease, autoimmune encephalitis, infectious disease

## Abstract

As the predominant antibody isotype at human mucosal surfaces, immunoglobulin A (IgA) exerts critical functions in mucosal defense and systemic immune homeostasis. The two IgA subclasses, IgA1 and IgA2, possess distinct structural and functional features and assemble into dimeric IgA (dIgA) and secretory IgA (sIgA) under physiological conditions. With advances in cryo-electron microscopy, the molecular architecture and assembly of sIgA have been clearly characterized. While IgA protects hosts from pathogenic infection and sustains immune balance, aberrantly altered IgA contributes to the pathogenesis of IgA nephropathy and autoimmune encephalitis. To date, IgA-based therapeutics have displayed promising therapeutic efficacy against respiratory disorders, inflammatory bowel diseases and oral fungal infections. Through standardized retrieval and categorical sorting of the published literature covering IgA structure, physiological functions, disease pathogenic mechanisms and therapeutic research, this review systematically organizes relevant research advances, analyzes the bottlenecks and translational prospects of IgA-targeted therapeutics, and provides objective references to accelerate the clinical translation of IgA antibody agents.

## 1. Introduction

Of the major antibody types (A, D, E, G, and M), IgA is uniquely specialized to undergo secretory transport and execute protective immune functions at mucosal sites [1]. In serum, IgA ranks second in abundance, accounting for 10–20% of total serum immunoglobulins and only inferior to IgG. Unlike other Ig classes, IgA exists in multiple molecular forms. IgA has two subclasses, IgA1 and IgA2, which differ in hinge region length, O-glycosylation patterns and tissue distribution [2]. Such subclass diversification is not found in IgG or other immunoglobulin isotypes. Binding of the J chain and IgA or IgM to PIGR activates uptake and transcytosis by epithelial cells, but IgA and IgM differ substantially in how they bind PIGR. IgM binds transiently, and IgA is covalently linked via disulfide bonds [3,4]. Monomeric IgA circulates in blood similar to serum IgG, whereas only J chain-linked dIgA can be transcytosed across epithelial cells via pIgR to form sIgA bound with secretory component (SC), a transport pathway exclusive to IgA. Early electron microscopy (EM) defined the double-Y morphology of sIgA dimers, Attachment of SC does not seem to change the overall length of the joined Fc regions. The idea that the domains of SC interact chiefly with the Fc regions of the dimer is supported by models based on solution structure analysis [5,6]. And cryo-electron microscopy (cryo-EM) has further resolved the atomic structure of the sIgA-Fc dimer and its assembly mechanism.

Different from IgG which frequently initiates strong pro-inflammatory responses to clear systemic pathogens, IgA exerts unique bidirectional immunomodulatory effects [7,8]. It preserves mucosal homeostasis by neutralizing pathogens without provoking excessive inflammatory injury, and at the same time triggers innate immune cell clearance through the IgA-specific receptor FcαRI [9]. Aberrant O-glycosylation of IgA1 triggers IgA nephropathy, an immune disease closely specific to IgA dysfunction, and IgA autoantibodies can also induce autoimmune encephalitis. In contrast to conventional IgG therapeutics with limited mucosal penetration, IgA exhibits prominent advantages in mucosal targeting and neutrophil activation, making it a promising candidate for treating respiratory disorders, inflammatory bowel diseases and oral candidiasis. Nevertheless, its clinical application is limited by molecular instability, technical bottlenecks in production and insufficient preclinical research.

Set against the research background of well-developed IgG-based antibody drugs, this review systematically illustrates the unique structural features, subclass properties, physiological functions and pathogenic mechanisms of IgA, as well as the latest advances of IgA therapeutics via diverse administration routes. It also analyzes current challenges and future directions to promote the clinical translation of IgA-based drugs.

## 2. The Structure of IgA

**Figure 1:**
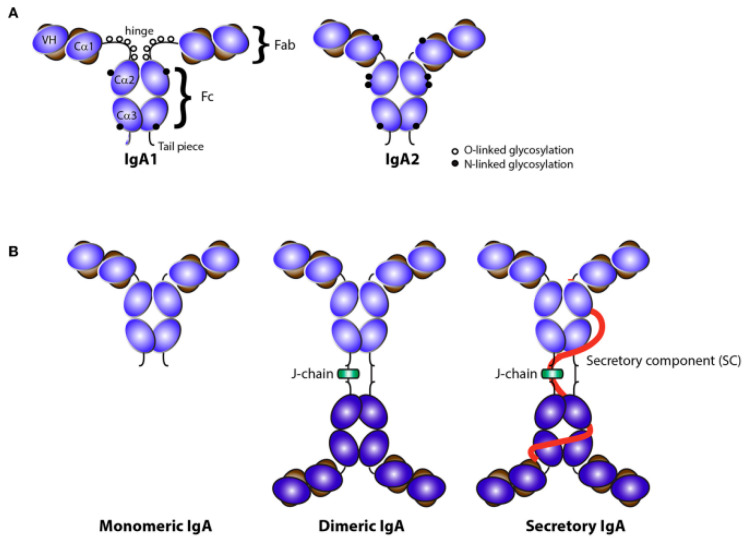
Structural features of IgA subtypes and their polymeric forms involved in mucosal immunity. Reprinted from Ref. [13], distributed under the Creative Commons Attribution (CC BY 4.0) License, copyright © 2019 Breedveld and van Egmond. Abbreviations: IgA, immunoglobulin A; IgA1/IgA2, immunoglobulin A subclass 1/2; Fab, fragment antigen-binding region; SC, secretory component; dIgA, dimeric IgA; sIgA, secretory IgA. (**A**) Comparison of monomeric IgA1 and IgA2 structures. IgA1 has a long hinge with O-linked glycosylation, while IgA2 has a truncated hinge lacking this modification. (**B**) Three forms of IgA: monomeric IgA; dIgA linked by a J chain; sIgA with an additional SC bound to dIgA.

A distinguishing feature of the two genetically and structurally related IgA subclasses, IgA1 and IgA2, is the extended hinge region in IgA1, which contains 13 additional amino acid residues compared with IgA2. This structural extension confers greater flexibility and enables recognition of spatially separated or conformationally constrained epitopes (depicted in Figure 1). The IgA monomer adopts a canonical immunoglobulin fold, consisting of two identical heavy (α) chains and two light chains; each α-chain comprises one variable domain (VH) and three constant domains (Cα1, Cα2, and Cα3). IgM and IgE possess four heavy-chain constant domains and lack a canonical hinge segment, while IgG and IgD hinge regions do not carry clustered O-linked glycans. Distinct from all these isotypes, IgA bears abundant N-linked glycans within its Cα1 and Cα2 domains that account for approximately six percent of its total molecular mass. Further setting IgA1 apart from every other antibody class is its elongated hinge region decorated with heterogeneous mucin-type O-linked glycans assembled from N-acetylgalactosamine, galactose, and sialic acid, a structural feature exclusive to IgA1 [10,11,12]. In vivo, serum IgA mainly exists as a monomer, while IgA primarily acts as polymers at mucosal sites. dIgA is stabilized via covalent bonding between two monomers bridged by the J chain. sIgA is the most abundant immunoglobulin in external secretions including saliva, tears, colostrum and intestinal fluid. Only IgA and IgM rely on J chain for polymerization, yet sIgA’s SC-mediated mucosal stability is exclusive to IgA mucosal defense. Although dimeric IgA constitutes the vast majority of sIgA, minor amounts of tetrameric or monomeric forms may also be present, with relative abundance varying by anatomical site and secretion type. Functionally, the Fc region encompassing the Cα2 and Cα3 domains mediates interactions with key receptors—including FcαRI (CD89) on myeloid cells and the pIgR during transepithelial transport—thereby supporting IgA’s dual roles in immune exclusion, pathogen neutralization, and anti-inflammatory homeostasis at barrier surfaces [2].

**Figure 2:**
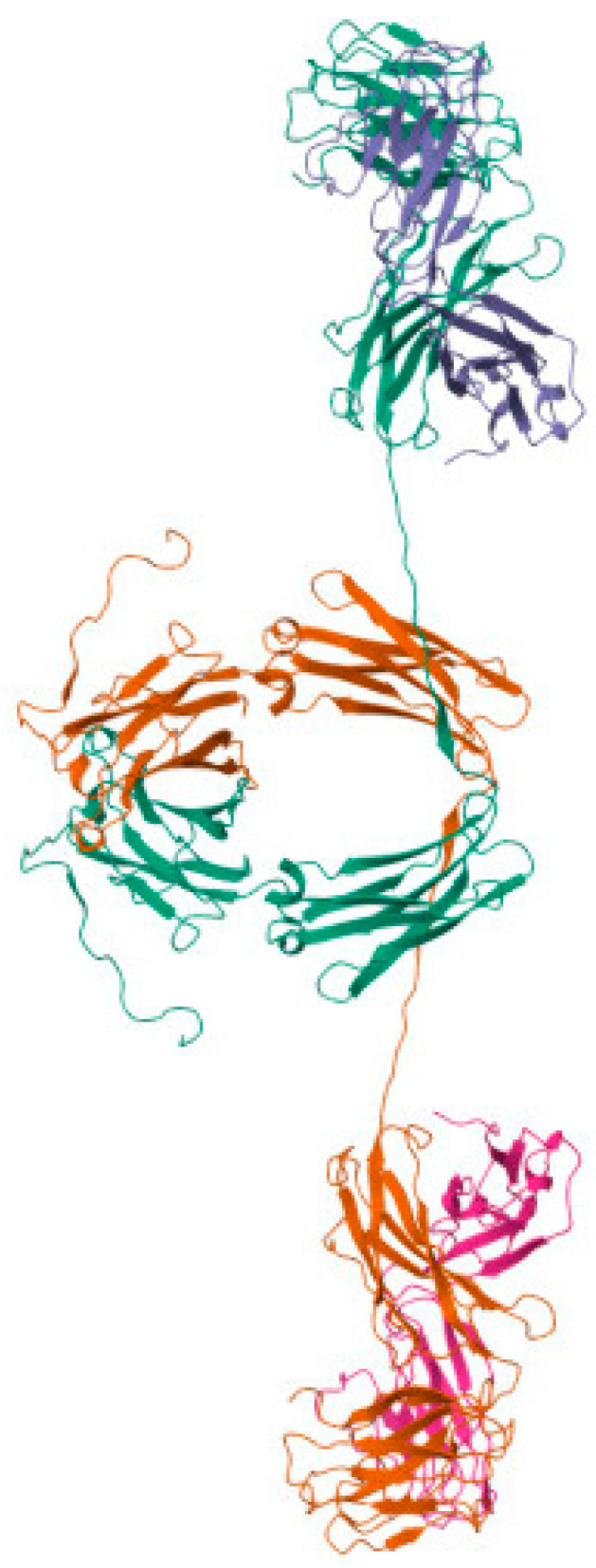
Full-length three-dimensional solution structure of monomeric human IgA1 (top Fab: purple & green; bottom Fab: orange & magenta; hinge: green; central Fc: orange & green) determined by solution scattering analysis (PDB ID: 1IGA). Abbreviations: IgA1, immunoglobulin A subclass 1; Fab, fragment antigen-binding region; Fc, fragment crystallizable region; PDB, Protein Data Bank.

The classic full-length solution structure of monomeric IgA1 (depicted in Figure 2) reveals its defining 13-residue longer hinge rich in Ser, Thr and Pro, decorated with 3–6 GalNAc/Gal/NeuAc O-glycans absent in IgA2’s short hinge. This extended glycosylated spacer grants IgA1 Fab arms high rotational flexibility and broader antigen cross-linking, yet makes it vulnerable to IgA1-specific bacterial proteases, whereas compact, glycan-free IgA2 hinge confers protease resistance for intestinal mucosal residence. The IgA1 Fc contains a conserved Asn263 N-glycosylation site governing FcαRI affinity; though IgA1 and IgA2 Fc domains share 94% sequence identity and similar dimeric assembly via J chain, their divergent hinge architectures drive differences in tissue distribution, stability and pathogenic roles like IgA nephropathy [14].

**Figure 3:**
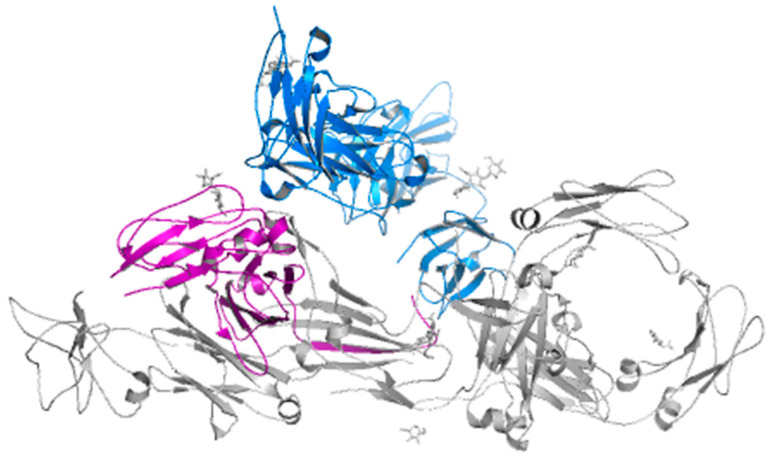
3D structure of human IgA-Fc dimer (gray) in complex with J chain (purple) and SC (blue) determined by cryo-EM analysis (PDB ID: 6UE7). Abbreviations: IgA, immunoglobulin A; Fc, fragment crystallizable region; SC, secretory component; cryo-EM, cryo-electron microscopy; PDB, Protein Data Bank.

The two IgA molecules in SIgA are linked via Cys471-mediated disulfide bonds to the J chain Early EM studies revealed that purified sIgA dimers, composed of two 7S subunits, adopt a double “Y” morphology [15]. In recent years, an increasing amount of attention has been given to IgA as a novel therapeutic antibody. However, despite extensive studies, sIgA structures have remained elusive. Solution scattering studies suggest that the two Fcα do not dock to each other in a straight manner in the IgA dimer, but adopt a slightly bent end-to-end arrangement [16]. The Fcα dimer forms a boomerang shape, analogous to part of the pIgM structure. Tailpiece β-strands (four in total, two from each Fcα) bundle to mediate dimerization [17]. Cryo-EM has enabled the determination of sIgA-Fc dimer structures at atomic resolution. These first-of-their-kind structures reveal pIgA in complex with the J chain, elucidate the mechanisms of polymer formation, and define the molecular basis for pIgA recognition by the pIgR SC (Figure 3) [18]. sIgA1 forms a tail-to-tail planar dimer, in which the two Fc regions are arranged at an angle of approximately 110° and tightly stabilized by the J chain, acting as a molecular clasp. The SC engages in extensive interactions with both Fcs and the J chain, binding diagonally across the ~50° gap between the two monomers. The core dimeric structure of sIgA1 is largely preserved in the sIgA2m2 tetramer and pentamer, consistent with the 94% sequence identity shared between the Fc regions of the two isotypes [17,18,19,20,21].

## 3. The Biofunction of IgA

### 3.1. The Physiological Functions and Mechanisms of IgA

**Figure 4:**
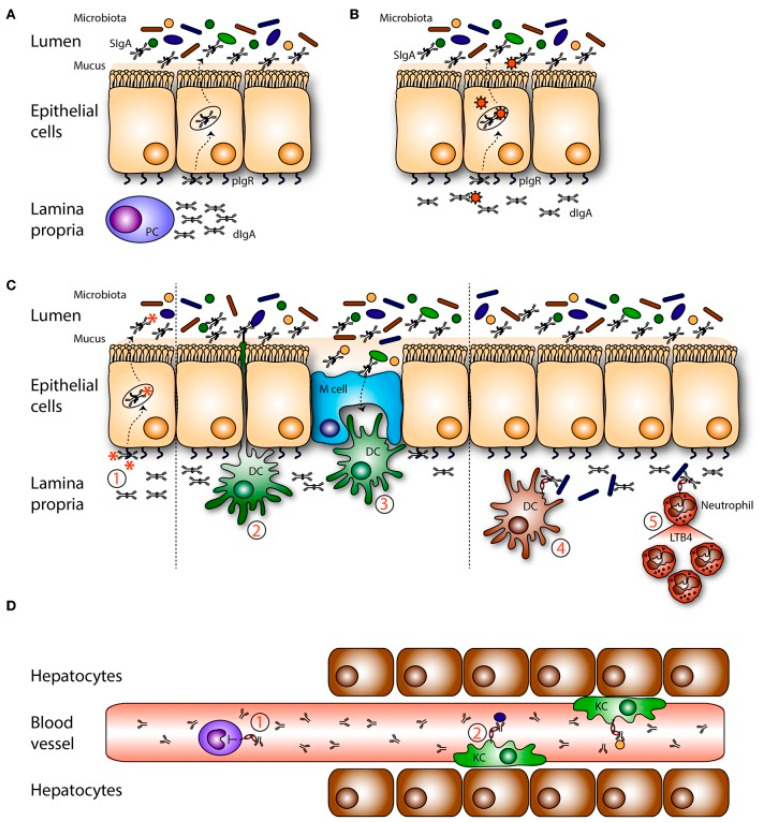
Diverse functions of IgA in barrier immunity and immune homeostasis. Reprinted from Ref. [13]. (**A**) sIgA-mediated immune exclusion; (**B**) intracellular neutralization of IgA during transcytosis; (**C**) mucosal antigen neutralization and pro-inflammatory responses induced by IgA; ➀ Infiltrated antigens and pathogens are opsonized by dIgA and transported back into the lumen. Sub epithelial DCs can ➁ sample antigens or ➂ take up SIgA-coated pathogens that enter via M cells. Pathogens in the lamina propria are coated with dIgA after which this immune complex is taken up by FcαRI- expressing ➃ DCs, and ➄ neutrophils. In response neutrophils secrete LTB4, hereby attracting more neutrophils, which will clear the infection. (**D**) systemic IgA functions in immune regulation and pathogen clearance. Serum IgA is ➀ capable of inhibiting (unwanted) pro inflammatory responses in the circulation via ITAMi signaling in monocytes. ➁ IgA-opsonized bacteria which have leaked into the circulation are taken up by FcαRI-expressing KC in the liver. Abbreviations: IgA, immunoglobulin A; sIgA, secretory IgA; dIgA, dimeric IgA; LTB4, leukotriene B4; DCs, dendritic cells; M cells, microfold cells; KC, Kupffer cells.

IgA serves as the principal antibody of the mucosal immune system. It establishes a multifaceted mucosal defense barrier through pathogen neutralization, inhibition of microbial adhesion to epithelial surfaces, and immune exclusion. The dIgA, predominantly synthesized by plasma cells in the mucosal lamina propria, is actively transported across the epithelium via the pIgR. During transcytosis (Figure 4A), dIgA associates with SC—a cleaved extracellular domain of pIgR—yielding stable sIgA, which is subsequently released into mucosal secretions of the respiratory, gastrointestinal, and urogenital tracts. SC not only facilitates transepithelial transport but also confers protease resistance, thereby enhancing sIgA stability and extending its functional half-life in harsh mucosal environments. Functionally, sIgA impedes pathogen colonization via steric hindrance and receptor blockade, neutralizes toxins and viral antigens, promotes microbial agglutination and entrapment for mucociliary or peristaltic clearance, disrupts biofilm formation, and reinforces mucin barrier integrity. Notably, sIgA exhibits low affinity for C1q and thus fails to efficiently activate the classical complement pathway; consequently, it mediates pathogen elimination with minimal induction of pro-inflammatory responses and negligible host tissue damage. IgA exists as two subclasses—IgA1 and IgA2—with distinct anatomical distributions and functional adaptations. IgA1 predominates in upper respiratory and nasopharyngeal mucosa, whereas IgA2—characterized by enhanced resistance to bacterial proteases owing to its unique hinge-region glycosylation—is enriched in the intestinal lumen. Their complementary expression ensures robust, context-dependent mucosal immunity and contributes critically to barrier integrity and immune homeostasis. Beyond luminal defense, dIgA mediates intracellular neutralization during pIgR-mediated transcytosis (Figure 4B), intercepting viruses and toxins within epithelial cells prior to their release into the lamina propria [22]. Furthermore, sIgA-opsonized pathogens may be retrotransported into the intestinal lumen for expulsion (Figure 4C). Subepithelial dendritic cells can sample antigens directly or internalize sIgA-coated microbes via microfold (M) cells to initiate localized adaptive immunity. In contrast, dIgA–pathogen complexes retained in the lamina propria engage FcαRI (CD89) on neutrophils, triggering recruitment, activation, and leukotriene B4 (LTB4) secretion, thereby amplifying local antimicrobial effector responses. Systemically, serum IgA contributes to immune regulation (Figure 4D). Circulating monomeric IgA engages FcαRI on hepatic Kupffer cells to facilitate clearance, while monomeric IgA binding to FcαRI on myeloid cells delivers an inhibitory signal via an immunoreceptor tyrosine-based inhibitory motif (ITAMi) that dampens excessive inflammation. Conversely, polymeric IgA or IgA-containing immune complexes induce FcαRI-dependent pro-inflammatory functions—including phagocytosis, reactive oxygen species production, and neutrophil extracellular trap (NET) formation—and preferentially activate the lectin complement pathway. Pathogens have evolved divers immune evasion strategies against IgA, including the expression of IgA-binding proteins and secretion of IgA1-specific proteases. Aberrant galactose-deficient glycosylation of IgA1 leads to autoantibody production and pathogenic immune complex deposition, events central to the pathogenesis of IgA nephropathy. Collectively, these findings underscore the dual role of IgA in maintaining mucosal tolerance and systemic immune homeostasis, and highlight its promising therapeutic potential against infectious and oncologic diseases [2,13].

### 3.2. The Pathological Role and Mechanism of IgA

#### 3.2.1. IgA-Mediated Organ-Specific Autoimmune Disorders

Under physiological conditions, secretory IgA dominates mucosal immune defense, maintaining mucosal and systemic immune homeostasis with minimal pro-inflammatory tissue injury. However, pathological conditions trigger structural defects, disturbed glycosylation, abnormal synthesis and impaired clearance of IgA, reversing its protective function and turning IgA into a key pathogenic driver. Dysregulated IgA participates in diverse immune diseases: abnormal IgA1 hinge O-glycans cause autoimmune injuries targeting the kidney and central nervous system; meanwhile, disease-specific N/O-glycan profiles of IgA serve as common pathogenic markers for respiratory, intestinal and rheumatic inflammatory disorders. IgA nephropathy (IgAN), the most prevalent primary glomerulonephritis across the globe, is an autoimmune renal disorder characterized by renal tissue damage induced by the deposition of circulating galactose-deficient IgA1 (Gd-IgA1)-IgG immune complexes. Its pathogenesis abides by the classic four-hit hypothesis: driven by the combined effects of genetic susceptibility and mucosal immune dysregulation, Gd-IgA1 bearing aberrant O-glycosylation within the hinge region is synthesized and secreted into the bloodstream, which markedly increases its serum concentrations (Hit 1) [23]; this aberrant IgA1 serves as an autoantigen and triggers the generation of specific IgG autoantibodies (Hit 2); Gd-IgA1 interacts with IgG autoantibodies to form pathogenic circulating immune complexes that are resistant to physiological clearance mechanisms (Hit 3); and ultimately, these immune complexes deposit in the glomerular mesangium and engage with mesangial cell surface receptors, thereby activating mesangial cells to undergo proliferative changes, accumulate excessive extracellular matrix, secrete abundant pro-inflammatory cytokines, and initiate the alternative complement pathway activation [24,25]. All these pathological cascades ultimately contribute to glomerular structural damage, tubulointerstitial inflammation and renal interstitial fibrosis. Beyond renal lesions, pathological IgA also disrupts systemic immune tolerance, amplifies chronic inflammatory responses, and drives progressive renal function deterioration, ultimately leading to chronic kidney disease and even end-stage renal failure in severe cases [26]. There is currently no disease-specific therapy and 30–40% of patients progress to kidney failure that reduces life expectancy by about 10 years [27]. Apart from the canonical four-hit cascade, long-term cohort studies establish aberrant IgA glycosylation as an independent predictor of progressive renal fibrosis and end-stage renal disease (ESKD) [26]. A UK registry of over 2400 IgAN patients reported half-developed kidney failure or death, with median renal survival of only 11.4 years. Even patients meeting the KDIGO proteinuria target (<1 g/d) carry substantial long-term risks: 20% of those with time-averaged proteinuria <0.44 g/g and 30% with 0.44–0.88 g/g progress to kidney failure within a decade [28]. A Chinese cohort of 1155 patients further validated that persistent proteinuria accelerates renal function decline, with a stricter target of <0.5 g/d linked to better 20-year renal survival [29]. Collectively, these data demonstrate that chronic IgA-derived immune injury causes lifelong cumulative kidney damage, and standard therapies rarely reach the eGFR protective threshold.

Henoch–Schönlein purpura (HSP, IgA vasculitis) is an IgA-dominated small-vessel vasculitis sharing core pathogenic cascades with IgAN. Emerging evidence points to a possible role of IgE in HSP, especially in pediatric patients with recurrent infections but no clear allergic sensitization [30]. Clinical data from a 5-year pediatric cohort [31] revealed elevated serum IgA in 54.34% of 138 patients, while infection-related IgE upregulation amplifies vascular permeability. Pathogenic IgG autoantibodies recognize galactose-deficient IgA1 (Gd-IgA1) to form tissue-depositable immune complexes, triggering perivascular inflammation. Comparable Gd-IgA1-IgG complex lesions and IgA glycosylation defects exist in both HSP and IgAN [32]. Multiple cohort and mechanistic works confirm graded pathological links between abnormal IgA and HSP onset, visceral involvement and relapse [33,34]. This and other studies showing varying degrees of IgA participation in disease pathogenesis deserve sufficient discussion.

The core pathogenic mechanism of IgA antibody-related autoimmune encephalitis is that IgA subtype autoantibodies specifically target NMDA receptor (NMDAR) on the surface of neurons, which can induce significant internalization and degradation of NMDAR, reduce the expression level of receptor by about 90%, and reduce the expression of synapsis-associated proteins simultaneously. This leads to a 90% reduction in NMDAR-mediated synaptic current. Patient serum reduced the frequency and amplitude of spontaneous excitatory postsynaptic currents (sEPSC) in primary hippocampal neurons by 75% (4.0 ± 1.5 Hz vs. 1.0 ± 0.4 Hz, *p* = 0.02) and 30% (57.4 ± 6.2 pA vs. 40.1 ± 7.9 pA, *p* = 2.3 × 10^−50^) suggesting extensive synaptic conduction dysfunction [35]. Furthermore, pathogenic IgA can bind to the FcαRI (CD89) on the surface of myeloid cells and microglia, activating the FcαRI-Syk-NF-κB signaling axis, which leads to the massive release of downstream pro-inflammatory factors such as TNF-α, IL-1β, and IL-6, thereby triggering chronic neuroinflammatory responses in the central nervous system [36,37]. The single-antigen-targeting IgA can induce the ITAMi inhibitory signal mediated by FcαRI, recruit SHP-1 phosphatase, and downregulate the excessive activation of myeloid cells, thereby exerting dual anti-inflammatory and neuroprotective effects. The classic immunomodulatory pathways of intravenous immunoglobulin (IVIG) include blocking activated Fc receptors, competitively occupying antigen-binding sites, accelerating the clearance of pathogenic antibodies, inhibiting complement activation and the release of inflammatory factors, and further alleviating neuroimmune damage [38].

In recent years, the latest research on IgA antibody-related autoimmune encephalitis has further refined the pathogenic mechanism and treatment system. In terms of mechanism, the specific synthesis of IgA in the sheath has been confirmed to be significantly associated with disease severity, cognitive impairment and recurrence risk, suggesting that central local immune activation is a key link in disease progression. At the diagnostic level, the fixed cell-based assay is a standardized method for detecting autoantibodies in the nervous system [39]. Determination of IgA-NMDAR antibodies could be used as a serologic test to uncover this autoimmune mechanism in patients with slowly progressive cognitive decline. The detection of autoantibodies can enhance the recognition rate of autoimmune encephalopathies related to dementia and mental disorders [40]. In the field of treatment, plasma exchange can rapidly eliminate pathogenic IgA antibodies, significantly reducing the serum anti-NMDAR-IgA titer from 1:32,000 to 1:1000, and rapidly reversing NMDAR internalization and synaptic damage, restoring synaptic protein expression and improving neurological deficits [35]. Monoclonal antibodies targeting FcαRI and novel formulations that induce ITAMi inhibition of signaling have entered preclinical research, and are expected to achieve long-lasting and precise anti-inflammatory effects without relying on hormones or cytotoxic drugs [36]. Based on the above mechanism and the latest progress, the targeted IgA treatment strategy can exert its effects in two aspects: protecting the synapses and inhibiting inflammation. On one hand, it can restore the expression of NMDAR and synaptic conduction function; on the other hand, it can block the excessive activation of the IgA-FcαRI-Syk-NF-κB pathway, alleviating neuroinflammatory damage. Novel patient-tailored targeted immunotherapies selectively interfere with pathogenic immune cascades, which could optimize long-term clinical outcomes and avoid broad adverse effects induced by long-term corticosteroid administration [41].

#### 3.2.2. Aberrant IgA Glycosylation in Multiple Inflammatory Diseases

Site-specific N-glycans on IgA1/IgA2 and unique O-glycans on IgA1 hinge determine IgA immune activity, and aberrant IgA glycosylation acts as a shared pathogenic marker across multiple disorders. As the first comprehensive plasma IgA glycomic comparison between severe SARS-CoV-2 and Influenza A infections conducted by Potaczek et al., researchers confirmed a shared ARDS-associated IgA1 O-glycan shift of reduced sialylation and elevated galactosylation across both pathogen groups, while COVID-19 generates infection-specific N-glycan signatures absent in influenza patients. Specifically, IgA2 N47 sites display diminished sialylation and bisection, and the shared N340/N327 sites of IgA1/2 show reduced fucosylation and antennarity alongside increased non-complex glycan abundance. These disease-specific IgA glycosylation profiles correlate with elevated plasma extracellular DNA and robustly drive neutrophil extracellular trap (NET) formation, thereby exacerbating pulmonary immunothrombosis in critically ill COVID-19 patients [42].

Florent Clerc et al. analyzed IgA1/IgA2 glycomics of 442 inflammatory bowel disease (IBD) patients and 120 healthy controls via LC-MS/MS. Significant intergroup disparities were observed in key glycan traits such as galactosylation, bisection, sialylation and antennarity. Crohn’s disease patients exhibited more pronounced deficiencies in IgA N-galactosylation, N-sialylation and IgA1 O-GalNAc abundance compared with UC patients. A predictive statistical model built on IgA glycomic signatures effectively discriminates healthy individuals from IBD patients with an AUC value of 0.79, eliminating the demand for invasive intestinal biopsies. This work extends existing mechanistic insights into CD and UC, and facilitates the development of non-invasive serological biomarkers to improve stratified patient management [43].

Mayboroda et al. combined IgG, IgA and TSNG glycomic data from the pregnancy-induced amelioration of rheumatoid arthritis (PARA) cohort. Rheumatoid arthritis (RA) patients exhibited dramatic depletion of high-galactose and high-sialic acid O-glycans on IgA1 hinge regions. The diagnostic model built solely on IgA glycan traits achieved an AUC of 0.896, outperforming IgG (0.852) and total serum N-glycome (TSNG) (0.871). The integrated multi-glycan signature reached an AUC of 0.945, largely independent of inflammatory DAS28 scores [44]. Overall, disease-specific IgA glycosylation signatures uncover inflammatory mechanisms and provide non-invasive biomarkers for respiratory, intestinal and autoimmune diseases (see Table 1).

## 4. Therapeutic IgA Drugs

IgA is an emerging therapeutic antibody candidate with distinct advantages over IgG for the treatment of inflammatory lung disorders, severe infections and tumors, stemming from its unique properties of mucosal targeting, potent neutrophil activation and bidirectional immunomodulation. An overview of different IgA therapeutic strategies categorized by administration route is presented in Table 2. Neutrophils represent the most abundant immune effector cell subset in humans, constituting 50–70% of peripheral blood leukocytes. These cells readily infiltrate lung infection foci, inflammatory lesions and tumor microenvironments. However, conventional IgG antibodies fail to efficiently trigger neutrophil cytotoxic effector functions. IgA potently elicits antibody-dependent cell-mediated cytotoxicity (ADCC), phagocytosis and immunomodulation through binding to neutrophil surface-expressed FcαRI, and exhibits markedly enhanced target cell clearance capacity relative to IgG. Furthermore, IgA promotes neutrophil recruitment to lesion sites by inducing LTB4 secretion, thereby augmenting local clearance of pathogens and tumor cells [45]. In inflammatory lung disorders including chronic obstructive pulmonary disease (COPD), asthma, severe pneumonia, and COVID-19, mucosal IgA displays reduced abundance or functional defects. Therapeutic IgA can restore the functionality of the pIgR/sIgA axis and suppress excessive neutrophil activation and NET formation, thereby attenuating pulmonary inflammation and tissue damage. In infectious diseases, IgA acts as a primary mucosal immune barrier that efficiently mediates pathogen agglutination and neutralization and confers resistance to degradation by bacterial proteases. Mucosa-resident respiratory IgA can markedly reduce infection susceptibility and shorten viral shedding duration [46].

IgA possesses unique core therapeutic advantages. First, it exhibits prominent mucosal tropism, which enables targeted accumulation at mucosal inflammatory lesions such as the respiratory tract to achieve precision therapeutic outcomes. Second, the distinct myeloid cell-activating signature mediated by IgA overcomes the key limitation of IgG, which is incapable of recruiting abundant peripheral neutrophils. Third, its bidirectional immunomodulatory capability enables pro-inflammatory responses against pathogens and tumors and suppresses aberrant immune activation to maintain mucosal immune homeostasis. With the continuous progress of antibody engineering, therapeutic IgA has become a promising developmental direction for antibody-based therapeutics. Moreover, IgA-initiated signaling does not rely on IgG-related Fcγ receptors, which enables it to bypass therapeutic resistance to IgG-based regimens. Additionally, IgA fails to trigger the classical complement cascade, thus reducing the incidence of immune-related adverse events [47].

### 4.1. Nasal Spray/Inhalation Administration

#### 4.1.1. Research and Technology Development on Respiratory Disease Adaptability

In 2019, Saito et al. established a facile strategy to generate recombinant human monomer-derived tetrameric monoclonal secretory IgA (SIgA). The target protein was successfully produced via co-expression of human IgA α heavy chains, light chains, joining (J) chains, and SCs in mammalian cell lines. The investigators compared the binding capacity and antiviral functional profiles of broadly neutralizing antibodies (bnAbs) harboring three distinct polymeric conformations—namely monomeric IgA, dimeric SIgA, and tetrameric SIgA—in the context of influenza A virus recognition and neutralization. Functional assays revealed that SIgA polymerization substantially enhances antibody neutralization efficacy against low-affinity influenza strains, while exerting negligible effects on the neutralizing potency of antibodies targeting high-affinity viral variants. In summary, SIgA polymerization expands the antigenic recognition spectrum of anti-influenza bnAbs without elevating maximal antiviral activity, thereby providing a technical framework for developing IgA-based therapeutic candidates targeting respiratory viral pathogens [48]. In 2020, Wu et al. established a versatile platform enabling the rapid isolation of fully human single-domain antibodies based on the IGHV3-66*01 germline framework, which conferred superior biophysical characteristics comparable to conventional camelid nanobodies. Biopanning screens against the SARS-CoV-2 spike receptor-binding domain (RBD) and S1 subunits identified antibodies recognizing five discrete epitopes. Group A antibodies possessed high binding affinity (0.6 nM) yet failed to exhibit neutralizing capacity, whereas Groups D and E exerted potent neutralizing activity against both pseudotyped and authentic SARS-CoV-2 viruses, with half-maximal inhibitory concentration (IC50) values ranging from 2.6 to 26.6 μg/mL. Notably, combinatorial treatment with D and E antibodies produced synergistic neutralizing effects, lowering the IC50 to a minimum of 0.51 μg/mL. Group D antibodies bound a cryptic epitope located at the trimeric interface of the spike protein without interfering with ACE2 receptor engagement. Furthermore, the SARS-CoV-2 RBD displayed a unique immunogenic signature that differentiates it from homologs of SARS-CoV and MERS-CoV. Collectively, this study provides critical insights and actionable targets for the rational design of sIgA-based therapeutic antibodies against respiratory coronaviruses [49]. In the same year, Ejemel et al. described the identification and functional characterization of a cross-neutralizing human IgA monoclonal antibody, named as MAb362. This antibody specifically recognizes the receptor-binding domain (RBD) of the SARS-CoV-2 spike protein and abolishes viral attachment and subsequent engagement with the host ACE2 receptor [50]; In 2022, Kanagaratham et al. demonstrated that allergen-specific sIgA attenuates IgE-mediated degranulation and activation of mast cells and basophils, as well as subsequent type I hypersensitivity reactions. These findings indicate that sIgA acts as a physiological immunomodulator to sustain mucosal immune homeostasis, supporting the therapeutic applicability of sIgA in the treatment of allergic respiratory disorders [51]. In the same year, Sun et al. engineered a bispecific single-domain secretory IgA (sIgA) antibody, named as bn03, which targets two conserved epitopes on the SARS-CoV-2 spike RBD. This bispecific antibody exhibited 4-fold enhanced neutralizing potency relative to its parental counterparts, with an IC50 of 0.28 μg/mL against wild-type SARS-CoV-2 and 0.11–0.76 μg/mL against emerging viral variants. Upon inhaled delivery, bn03 efficiently accumulated within pulmonary tissues and diminished lung viral loads by 2.9–3.6 log units in mice, conferring robust, broad-spectrum protective efficacy against diverse SARS-CoV-2 variants [52]. In 2023, the same research team further resolved the cryo-electron microscopy structure of the bn03-SARS-CoV-2 spike protein complex and characterized highly conserved cryptic epitopes. These epitopes endow bn03 and analogous IgA therapeutics with potent broad-spectrum neutralizing capacity against prevalent circulating SARS-CoV-2 variants [53]. Bohländer et al. conducted a systematic review delineating the immunoregulatory roles of IgA and FcαRI within respiratory mucosal immune homeostasis and pulmonary inflammatory pathogenesis. This work establishes a fundamental theoretical framework for the development of IgA-centered therapeutic strategies to treat common respiratory inflammatory disorders, including asthma and COPD [46]. In 2024, Huang et al. established a versatile, high-yield sIgA expression platform. Their findings revealed that recombinant anti-SARS-CoV-2 sIgA persists within the respiratory tract and pulmonary tissues for more than 72 h post intranasal delivery with undetectable systemic leakage, confirming robust mucosal tropism toward respiratory tissues [54]. In the same year, Marcotte et al. demonstrated that IgG-to-secretory IgA (sIgA) molecular conversion restores mucosal neutralizing potency against Omicron subvariants and circumvents viral immune evasion. Compared with parental IgG counterparts, dimeric and secretory IgA confer elevated neutralizing potency against multiple circulating SARS-CoV-2 variants of concern [55]. In 2025, Zhang et al. validated that intranasally administered sIgA1 elicits markedly enhanced neutralizing efficacy against Omicron sublineages relative to homologous IgG. Specifically, sIgA1 neutralized the BA.2.76 subvariant with a 161.3-fold increase in potency compared with IgG. In hACE2 transgenic mouse models, intranasal sIgA1 achieved a protective efficacy of 96.9%, which was substantially higher than the 82.1% efficacy conferred by IgG, while displaying extended mucosal retention time and durable protective potency [56].

#### 4.1.2. Preclinical Exploration and Clinical Trial Progress

In recent years, research teams led by Zhong Nanshan and Chen Ling have conducted sequential investigator-initiated trial (IIT) studies on the intranasal COVID-19 vaccine NB2155. Clinical outcomes verified that two-dose immunization with NB2155 induced a 51.5-fold elevation in nasal sIgA levels, which substantially outperformed the 4.5-fold sIgA induction observed following single-dose vaccination. This robust mucosal immune response confers protection against symptomatic infection under a community infection rate of 85%, validating the capacity of intranasal administration to induce potent respiratory mucosal sIgA responses [57]. In 2025, the research group further published a mechanistic investigation in JCI Insight, demonstrating that intranasal booster vaccination facilitates memory B cell subset differentiation and mucosal trafficking. These findings unravel the core cellular mechanisms governing mucosal sIgA generation and provide fundamental theoretical underpinnings for the clinical translation of intranasal mucosal vaccine platforms [58]. Recently, Beukenhorst et al. reported in Science Translational Medicine that intranasal administration of the broad-spectrum influenza-neutralizing antibody CR9114 exhibited favorable safety and tolerability in a first-in-human Phase 1 trial, with a nasal residence half-life of 3 h. Compared with once-daily administration, twice-daily dosing yielded a 92-fold increase in mucosal trough antibody concentrations and provided robust protective efficacy against influenza viral challenge in nonhuman primate models, reinforcing the feasibility of mucosal passive immunization strategies [59].

### 4.2. Oral Administration

#### 4.2.1. Research and Mechanism of Intestinal Disease Adaptation

In 2016, Okai et al. reported that oral high-affinity monoclonal IgA W27 remodels gut microbiota and alleviates murine lymphoproliferative disease and colitis. W27 targets the EEHI motif of bacterial serine hydroxymethyltransferase (SHMT), selectively inhibiting Escherichia coli growth while showing no binding or growth suppression toward the probiotic Lactobacillus casei in vitro [60]. Oral IgA therapy confers therapeutic efficacy in murine models of lymphoproliferative disorders and colitis via modulation of intestinal microbiota composition, representing a promising strategy for the treatment of inflammatory bowel disease (IBD). In 2024, Tang Y et al. reported that administration of monoclonal IgA W27 attenuated intestinal inflammation and suppressed azoxymethane/dextran sulfate sodium (AOM/DSS)-induced colorectal carcinogenesis [61]. Moreover, accumulating evidence indicates that IgA deficient (IgA−/−) mice and mice lacking polymeric Ig receptor (pIgR−/−) are more susceptible to colitis, suggesting that intestinal IgA executes a pivotal protective role against colitis development [62].

#### 4.2.2. Advances in the Preclinical Development of sIgA-Based Therapeutics

In 2024, Wang et al. demonstrated that the human milk-derived sIgA candidate LCTG-002 exerted 6.3-fold greater inhibitory potency than control IgA toward SARS-CoV-2 spike–ACE2 binding, achieving a maximum inhibitory rate of 58% at a concentration of approximately 240 μg/mL. In K18-hACE2 mouse models, intranasal LCTG-002 administration markedly diminished pulmonary SARS-CoV-2 viral titers by up to 4.9 log units at dosages of 250 μg/day and 1 mg/day, validating milk-derived polyclonal sIgA as a viable mucosal therapeutic modality for respiratory viral pathogen infections. This preclinical investigation verified that LCTG-002 possesses high purity, potent bioactivity and favorable in vivo safety profiles, facilitating the translational development of milk-derived polyclonal sIgA candidates for SARS-CoV-2 and other mucosal infectious diseases [63]. Recently, Lactiga Therapeutics obtained financial support from the NIH to advance preclinical assessments of oral maternal antibody intervention against inflammatory bowel disease (IBD), which aims to characterize the immunomodulatory capacity of mucosal sIgA in chronic intestinal inflammatory pathologies [64].

### 4.3. Research Progress and Clinical Translation of Intravenous IgA-Containing Immunotherapeutics

Intravenous immunoglobulin (IVIg) confers robust immunomodulatory and anti-inflammatory properties in COVID-19 patients [65,66]. In 2021, Danieli et al. demonstrated that IVIg elicits potent immunomodulatory and anti-inflammatory activities in patients with severe COVID-19. IVIg mitigates cytokine storm pathogenesis via balancing pro- and anti-inflammatory cytokine profiles, suppressing the release of IL-6, TNF-α and other pro-inflammatory mediators, restoring systemic immune homeostasis and optimizing patient clinical outcomes. Specifically, early intervention with high-dose IVIg (>15–20 g/day) was correlated with reduced 28-day mortality, shortened ICU hospitalization duration, and decreased mechanical ventilation dependence among critically ill COVID-19 patients [67]. In 2022, Rahmel et al. conducted a multicenter cohort study, verifying that Pentaglobin, a plasma-derived human immunoglobulin formulation containing endogenous IgA, ameliorates clinical manifestations and alleviates pulmonary inflammatory damage in severe COVID-19 cases. This retrospective multicenter investigation enrolled 316 critically ill COVID-19 patients with 30-day mortality set as the primary endpoint. After adjusting for confounding factors via Cox regression and SuperLearner-based propensity score weighting, the study identified no statistically significant survival benefits in the overall cohort (HRadj: 0.83; 95% CI: 0.55–1.25; *p* = 0.374). However, two patient subgroups, including individuals free from mechanical ventilation at treatment onset (HRadj: 0.23; 95% CI: 0.05–1.08; *p* = 0.063) and those receiving daily doses of ≥15 g for a minimum of 3 consecutive days (HRadj: 0.65; 95% CI: 0.41–1.03; *p* = 0.068), exhibited marginal yet promising survival benefits. These observations suggest that timely administration and sufficient dosing regimens yield favorable therapeutic outcomes with acceptable safety profiles, although these preliminary findings necessitate further verification in prospective randomized controlled trials [68]. The clinical efficacy and safety of Pentaglobin have been rigorously validated via decades of clinical research and real-world clinical application. This biologic agent stands as the most clinically mature IgA-containing immunoglobulin formulation with robust cumulative clinical evidence [46]. Moreover, multiple ongoing Phase III clinical trials of Trimodulin targeting severe community-acquired pneumonia and moderate-to-severe COVID-19 further corroborate the translational potential of IgA-based immunotherapies for treating life-threatening pulmonary infectious diseases [69,70].

## 5. Mechanistic Research on sIgA-Driven Oral Anti-Candida Immune Defense

In 2021, Ost et al. demonstrated that sIgA elicited by adaptive immune responses mediates symbiotic homeostasis between the host and oral Candida albicans, which not only represses fungal pathogenic transformation but also mitigates mucosal tissue damage triggered by excessive inflammatory responses. Intestinal sIgA specifically recognizes and restricts the formation of hyphal morphotypes as well as hypha-enriched Als1/Als3 adhesins expressed by Candida albicans. Relative to wild-type littermates, Rag1^−/−^ and μMT^−/−^ mice exhibited augmented hyphal proliferation and aggravated colonic tissue injury in DSS-induced colitis models. Conversely, host immune selection against fungal hyphae improves the commensal adaptability of C. albicans, and Als3-targeted NDV-3A vaccination confers robust protection against C. albicans-related intestinal pathological damage. Collectively, these findings verify that adaptive immunity sustains host-fungal symbiotic balance via uncoupling fungal mucosal colonization from pathogenic activation [71]. In 2024, Wang et al. reported that sIgA reduces cellular ergosterol accumulation in Candida albicans, thereby suppressing fungal hyphal differentiation and virulence activation. Notably, sIgA exerts markedly superior inhibitory potency against hyphal outgrowth compared with homologous IgG. Using ergosterol pathway-deficient fungal mutant strains (erg11Δ/Δ, erg3Δ/Δ, and erg11Δ/Δ erg3Δ/Δ), researchers observed abrogated sIgA-mediated hyphal suppression in all mutant backgrounds. Additionally, sIgA-induced inhibitory effects on oral epithelial cell adhesion were attenuated in erg11Δ/Δ and erg3Δ/Δ strains and completely abolished in double-knockout erg11Δ/Δ erg3Δ/Δ strains, validating that the ergosterol biosynthetic pathway underpins sIgA-dependent hyphal repression. This study first identified that sIgA restrains C. albicans hyphal maturation and virulence by disrupting ergosterol biosynthesis, confirming ergosterol as a core modulator governing C. albicans-host epithelial crosstalk [72]. In 2025, Göritzer et al. optimized plant-based antibody expression platforms via combinatorial endoplasmic reticulum remodeling and chaperone overexpression, substantially elevating the production yield of recombinant antifungal sIgA and resolving critical restrictions constraining the scalable manufacturing of IgA-based immunotherapeutics. The research team validated that endoplasmic reticulum (ER) engineering in Nicotiana benthamiana significantly augments the production and structural assembly efficiency of anti-SARS-CoV-2 sIgA. Functional quantification revealed that ER-engineered plant lines yielded multimeric COVA2-15 sIgA1 at 6.7-fold, 5-fold, and 5.5-fold higher levels than wild-type counterparts, while 2E8 sIgA1 production was increased 3–4-fold. Combined with calnexin (CNX1) overexpression, the yield of COVA2-15 sIgA was further upregulated to 910 mg per kilogram of fresh plant tissue, representing a 16-fold elevation compared with wild-type controls. Meanwhile, the sIgA assembly efficiency was improved from 79.6% to 86.4% for COVA2-15 and from 63.1% to 74.1% for 2E8, addressing major technical barriers to the heterologous production of complex multimeric antibody isoforms in plant expression systems [73]. Furthermore, probiotic interventions can indirectly boost sIgA secretion and biological functionality by remodeling oral microbial communities, stimulating endogenous immunoglobulin synthesis and antimicrobial factor secretion, enhancing host mucosal immune resistance against opportunistic Candida albicans infection, and reducing the recurrence risk of oral fungal disorders, thus supporting the clinical translational potential of probiotic-assisted sIgA-targeted adjuvant immunotherapy [74].

## 6. Discussion

As an alternative natural intervention to IgA biologics, herbal preparations possess definite pharmacological activity in regulating IgA levels. Chen et al. discovered that Bao-Yuan tablet (BYT), a compound formula consisting of ginseng, astragalus, licorice and cinnamon bark, significantly lowered abnormally elevated serum IgA at an effective dose of 3 g/kg/day in spleen-qi deficient mice [75]. It is speculated that the excessive IgA secretion in SQD mice may stem from hyperactivation of the splenic TLR4/NF-κB pathway, and the 141 bioactive constituents identified from BYT could modulate this overactivated signaling axis to normalize disordered IgA secretion, providing a multi-target herbal strategy for IgA-related immune dysfunction. Such herbal regulators provide a complementary therapeutic perspective for correcting aberrant IgA glycosylation and overproduction in IgA-mediated inflammatory disorders, broadening the intervention spectrum for IgA-related immune dysfunctions beyond antibody biologics.

Accumulating preclinical and clinical evidence demonstrates that IgA mediates sophisticated, bidirectional immunomodulatory effects during host anti-pathogen defense and systemic inflammatory modulation. Updated functional validation approaches have further delineated two core biological functions of IgA: protection against invasive exogenous pathogens and preservation of host immune homeostasis. This dual capacity encompassing mucosal defense and immune regulation renders IgA a promising therapeutic modality for inflammatory pulmonary disorders. The clinical management of such diseases requires not only the blockade of recurrent mucosal infections and reinforcement of host mucosal barrier integrity, but also the attenuation of aberrant inflammatory cascades to sustain systemic immune homeostasis [46]. As the dominant antibody isotype within mucosal immune compartments, IgA harbors distinctive translational value in mucosal anti-infective prophylaxis, tumor immunotherapy and inflammatory disease modulation, primarily attributed to robust FcαRI-dependent antibody-dependent cellular cytotoxicity (ADCC) activity. Multiple inflammatory lung pathologies are characterized by dysregulated IgA expression and transmembrane trafficking, further corroborating the clinical feasibility of IgA-centered immunotherapies. Despite the substantial translational prospects, IgA-based therapeutics still face multiple inherent limitations, including short in vivo circulatory half-life, high aggregation propensity, elevated glycosylation variability, insufficiently validated preclinical model systems, and underdeveloped scalable manufacturing pipelines [47,76]. Multiple intrinsic drawbacks limit IgA clinical translation. IgA1′s long O-glycosylated hinge is cleaved by bacterial proteases, and galactose-deficient hinge glycans trigger IgA nephropathy [23,25]. Unlike IgG, IgA cannot bind FcRn and has a short serum half-life [2,76]. It scarcely activates classical complement. IgA delivers dual FcαRI signals: monomeric IgA suppresses inflammation, while polymeric IgA induces neutrophil-mediated tissue damage [37]. Nevertheless, cutting-edge antibody engineering strategies—including terminal sialylation remodeling, IgA2.0 variant construction, albumin-binding domain conjugation and site-directed mutagenesis—have progressively resolved technical bottlenecks regarding in vivo durable efficacy, molecular homogeneity and large-scale industrial production [77,78,79,80]. Meanwhile, optimization and standardization of humanized preclinical animal models facilitates accelerated preclinical translational progression. Collectively, engineered recombinant IgA derivatives are poised for broadened clinical applicability. Mucosal targeted delivery systems overcome the insufficient mucosal penetration capacity of conventional IgG therapeutics, enabling precise intervention against pulmonary inflammatory lesions and localized mucosal infections [81]. Furthermore, the superior anti-tumor potency of IgA overcomes the inherent functional restrictions of IgG, supporting therapeutic outcomes in both solid malignancies and autoimmune pathologies. Combination regimens integrating engineered and conventional polyclonal IgA formulations will further broaden the clinical indication spectrum of IgA-based immunotherapy [82,83]. Of note, numerous engineered IgA therapeutic candidates have entered successive clinical developmental stages. Looking ahead, alongside continuous technological optimization and accelerated translational progression, IgA-based biologics will serve as vital functional complements to conventional IgG therapeutics, establishing a diversified multi-isotype antibody therapeutic framework and ultimately providing innovative clinical intervention strategies for a wide range of infectious, inflammatory and neoplastic diseases.

## Figures and Tables

**Table 1:** IgA aberrations and pathogenic manifestations in related diseases.

Disease	Abnormal IgA Feature	Pathogenic Pathway	Implication	References
IgAN	Hinge O-galactose-deficient IgA1 (Gd-IgA1)	Gd-IgA1-IgG immune complex deposits in glomeruli	30–40% progress to renal failure within 10 years	[23,24,25,26,28]
HSP	Circulating Gd-IgA1-IgG complexes	Gd-IgA1-IgG form tissue-depositable immune complexes, triggering perivascular inflammation	Purpura, abdominal pain; high pediatric relapse risk	[30,31,32,33]
IgA autoimmune encephalitis	Pathogenic intrathecal anti-NMDAR IgA autoantibodies	IgA autoantibodies specifically bind neuronal NMDAR and trigger receptor loss; FcαRI-Syk-NF-κB drives neuroinflammation	Insidious cognitive decline, frequent recurrence	[35,36,37,38,41]
SARS-CoV-2/Influenza A	Deficient mucosal sIgA, disease-specific IgA glycosylation	Impaired mucosal defense, NET-mediated lung injury	Dyspnea, ARDS in severe cases	[42]
IBD	Reduced intestinal sIgA, Disease-specific IgA1/IgA2 glycan defects	Aberrant IgA glycosylation impairs mucosal homeostasis; gut barrier damage	Recurrent intestinal inflammation, cancer risk	[43]
RA	IgA1 hinge O-glycan galactose/sialic acid depletion	Dysregulated systemic inflammatory response	Symmetric joint pain; progressive joint destruction	[44]

Abbreviations: IgA, immunoglobulin A; IgAN, IgA nephropathy; HSP, Henoch–Schönlein purpura; Gd-IgA1, galactose-deficient IgA1; NMDAR, N-methyl-D-aspartate receptor; ARDS, acute respiratory distress syndrome; IBD, inflammatory bowel disease; RA, rheumatoid arthritis; NET, neutrophil extracellular trap.

**Table 2:** Overview of IgA therapeutic regimens by administration route.

Administration Route	Therapeutic Type	Adapted Diseases	Research Stage	Advantages	Limitations
Nasal/inhaled	Recombinant sIgA mAb	Influenza, COVID-19, asthma, COPD	Preclinical + Phase I (influenza CR9114)	Respiratory-specific distribution; few systemic side effects	Poor mucosal stability; hard mass production
Oral	Recombinant/milk-derived sIgA	IBD, intestinal infection, oral candidiasis	Preclinical	Local mucosal action, low systemic toxicity	Degraded by digestive/oral proteases, low bioavailability
Intravenous	IgA-containing IVIG (Pentaglobin; Trimodulin)	Severe COVID-19; pulmonary inflammatory damage	Clinically used; Trimodulin Phase III	Rapid broad immunomodulatory effect	No mucosal tropism, short half-life, infusion risks

Abbreviations: IgA, immunoglobulin A; sIgA, secretory IgA; mAb, monoclonal antibody; COPD, chronic obstructive pulmonary disease; IBD, inflammatory bowel disease; IVIG, intravenous immunoglobulin.

## Data Availability

No new data were created or analyzed in this study. Data sharing is not applicable to this article.
